# Application of a Robust Thermoelectric Gas Sensor in Firewood Combustion Exhausts

**DOI:** 10.3390/s23062930

**Published:** 2023-03-08

**Authors:** Gunter Hagen, Julia Herrmann, Xin Zhang, Heinz Kohler, Ingo Hartmann, Ralf Moos

**Affiliations:** 1 Department of Functional Materials, Zentrum für Energietechnik (ZET), University of Bayreuth, D-95440 Bayreuth, Germany; functional.materials@uni-bayreuth.de (J.H.); functional.materials@uni-bayreuth.de (R.M.); 2 Institute for Sensor and Information Systems (ISIS), Karlsruhe University of Applied Sciences, D-76133 Karlsruhe, Germanyheinz.kohler@h-ka.de (H.K.); 3 Deutsches Biomasseforschungszentrum (DBFZ), D-04347 Leipzig, Germany; ingo.hartmann@dbfz.de

**Keywords:** wood combustion, combustion control, FTIR analytics, exothermicity gas sensor, screen printed thermocouple

## Abstract

The quality of wood combustion processes can be effectively improved by achieving the automated control of the combustion air feed. For this purpose, continuous flue gas analysis using in situ sensors is essential. Besides the successfully introduced monitoring of the combustion temperature and the residual oxygen concentration, in this study, in addition, a planar gas sensor is suggested that utilizes the thermoelectric principle to measure the exothermic heat generated by the oxidation of unburnt reducing exhaust gas components such as carbon monoxide (CO) and hydrocarbons (C_x_H_y_). The robust design made of high-temperature stable materials is tailored to the needs of flue gas analysis and offers numerous optimization options. Sensor signals are compared to flue gas analysis data from FTIR measurements during wood log batch firing. In general, impressive correlations between both data were found. Discrepancies occur during the cold start combustion phase. They can be attributed to changes in the ambient conditions around the sensor housing.

## 1. Introduction

Biomass is considered as a CO_2_-neutral, regenerative energy source for heat and power generation. Especially, small wood log fireplaces are becoming increasingly popular. However, as the current status of firing technology emissions from furnaces operated in domestic households is that they significantly contribute to air pollution [1,2,3,4], applications such as catalysts for the abatement of air pollutants are needed [5,6]. Flue gas comprises a huge variety of components depending on several factors such as the used type of wood, its origin, its humidity, etc. In addition, the operation procedure itself is highly unique. As of today, harmful components such as particulate matter (PM), carbon monoxide (CO) and various hydrocarbons (HC) are emitted to the atmosphere without any exhaust gas aftertreatment. The further use of biomass will mainly depend on the successful development of technical solutions to lower the emissions of toxic gas components and particulate matter loaded with organic matter. Automated control of the combustion air is the starting point to reduce such emissions.

In case of single-room furnaces (log-fueled in the low power range), most applications are operated without automatic combustion air control, i.e., the combustion air is manually adjusted with the consequence that, in most situations, the combustion air streams are not well adapted to the actual combustion situation of a batch process. In contrast, scientific research to achieve much better combustion quality and lower emissions is at a much more advanced stage. Already in 1989, Nussbaumer et al. [7] published a study on the dependence of gaseous emissions of wood firing on its combustion temperature and on the influence of the residual oxygen concentration (ROC) in the exhaust gas. Furthermore, initial promising experiments from Butschbach et al. [8] showed that not only the combustion temperatures and ROC, but also the content of non-oxidized and partially oxidized exhaust gas components, should be considered by an advanced combustion air control system. The authors illustrated significant technological steps towards minimizing emissions. In 2018, it was shown that there is no strict correlation between the combustion temperature, ROC, and CO/HC content [9]. This means that all three parameters must be measured separately using sensors in order to describe whether the actual status of a batch wood combustion process is good enough, which is a prerequisite for the introduction of advanced combustion process control strategies. The authors showed possible emission reduction of CO by about 80% by use of an automated combustion air control system based on these three signals.

Whereas in situ sensors for long-term monitoring of temperature and ROC in exhaust gas environments are available, the availability of appropriate, long-term stable CO/HC gas sensors is the main obstruction for a widespread application of advanced automated combustion air control concepts. Suitable sensors must be able to withstand harsh conditions regarding aggressive gas components (such as organic acids, alcohols, aldehydes, and others) and fine PM loaded with condensated, high-boiling organic compounds and soot at temperatures up to 500 °C. Up to now, the robustness and stability of such sensors have been the subject of research. Furthermore, sensors ought to be inexpensive to ensure public acceptance.

In former studies, the sensitivity and signal stability of tin-oxide-based gas sensor arrays, as well as mixed-potential-type sensors were investigated, because these devices were considered as potential candidates for in situ CO/HC monitoring in wood combustion flue gases [10]. A recently published approach comprises a mixed-potential-type sensor based on solid electrolytes. The former problems with long-term stability have been solved by the modification of the Au,Pt-YSZ-mixed-potential electrode material and optimization of the operation temperature. In addition, the sensitivity can be checked in defined time periods of operation by electrochemical impedance spectroscopy (EIS) to the environmental air conditions of the place of operation, and a special strategy has been developed for sensor regeneration in cases of sensitivity loss. More details about this high-temperature mixed-potential gas sensor and its long-term behavior regarding the flue gas of a wood log fueled fireplace are reported in [11], and basic studies of the mixed-potential generation of Au,Pt-YSZ electrodes and a method of sensitivity check and stability improvement are published in [12,13].

Another promising principle is suggested in the present study. It comprises a thermoelectric sensor used to measure the reducing components in the flue gases from biomass combustion. Its simple setup achieves a long-term, stable and cost-effective operation. Secondary factors that affect the sensor signal can be resolved. Optimizations of the sensing element itself, as well as improvements concerning the sensor housing, led to reliable and reproducible results in the flue gas analysis.

## 2. Materials and Methods

In this section, the thermoelectric gas sensor is introduced in detail with respect to its setup, the measuring principle and typical parameters for its sensitivity. In principle, external factors contribute to the sensor signal, and therefore, represent a challenge to be overcome. It is shown that for sensor application in flue gas such circumstances can be overcome.

### 2.1. Sensing Element and Sensing Mechanism

**Figure 1:**
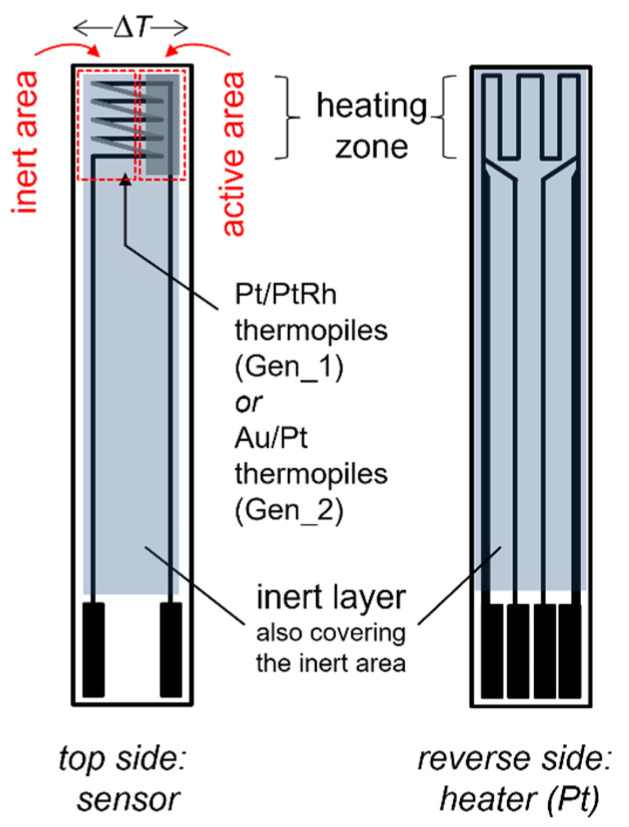
Sketch of the sensor setup to illustrate the measuring principle: A catalytically activated thick-film layer (“active area”) leads to a temperature increase due to exothermic oxidation of the reducing analytes. The screen printed thermopile structure measures the temperature difference of the inert area within the sensor tip as a voltage signal. The heating element on the reverse side provides the required sensor operation temperature.

In general, the sensor signal originates from a temperature increase generated by exothermic oxidation of the reducing gas components at a catalyzed functional layer (Figure 1). The tip of the ceramic sensing element comprises a catalyzed and a chemically inert area, so that a temperature gradient forms in between these both areas when reducing gas components are present. This temperature difference is measured by a special structure of serially connected thermocouples (thermopile) covered by both layers. Due to the Seebeck effect, a sensor signal in the µV range forms. This voltage directly depends on the temperature difference between the inert and the catalyzed areas, and therefore, has been experimentally confirmed linearly on the concentration of a reducing component in the test gas [14]. It should be noted here that the denotation “thermoelectric sensor” could be misleading, as the thermoelectric materials are just a means for measuring the temperature difference. The Seebeck coefficient of the materials is not affected by the analyte (in contrast to [15], the authors of which created direct thermoelectric sensors). A similar concept as that which is presented here (but not using thermopiles for temperature difference measurement) was introduced as a “calorimetric sensor” [16]. There, the exothermic heat generation under catalytic oxidation conditions of the un- or partly oxidized flue gas components on the functional layers are sensitively measured by resistive meanders forming a Wheatstone bridge structure in a microstructured calorimetric sensor chip. The signals of those chips, when they are applied to flue gas, would represent the total amount of such (toxic) gas components.

Figure 1 illustrates the sensor principle and its setup. The planar-sensing element is based on an alumina substrate (Rubalit 708, CeramTec). It is fully manufactured with thick-film technology. On the top side of the substrate, the functional layers are applied, covering the thermopile. Two different metal materials are alternatingly connected between both areas in the sensor tip, where the temperature difference generates the sensor voltage. The signal output can be increased by the number of serially connected thermocouples, and the difference in the Seebeck coefficients of both metals determine the voltage measured as the sensor signal. Screen printable pastes of Pt and PtRh (10% Rh, type S thermocouple) offer a good thermal stability [17]. However, stronger signals occur when one is using Au/Pt junctions due to their larger differences in the Seebeck coefficient [18]. It must be noted here that the Seebeck coefficient itself depends on the temperature. This has to be considered as well. The voltage is picked off by electrical feed lines of one of these materials. Therefore, the measured voltage is proportional to the temperature difference between both areas in the sensor tip and is primarily not affected by the temperature drop from the tip to the contacts. All the metal feed lines are protected by a glass ceramic cover (QM 42, DuPont), except in the “active” area. There, a noble metal-loaded porous alumina thick-film acts as a catalyst to enhance the oxidation of the gas species that are to be detected. The sensor operation temperature, as well as the catalyst material itself (such as surface, kind of precious metal and loading amount), have an influence on the reaction kinetics, and so, exothermic heat generation occurs in the activated area. One can even use such sensing elements as a tool for catalyst characterization by modulation of the heater temperature, as it was shown in [19].

In the present case, the functional layer was achieved as follows: We loaded alumina powder with 1 wt.% Pt (Gen_1 sensors) or 1 wt.% Pt with additional 1 wt.% Pd (Gen_2 sensors). Such powders were processed to become screen printable pastes, which were applied to the sensing element and sintered at 850 °C to achieve a porous morphology (details can be found in [19]).

As the thermal conductivity of the substrate material may reduce the temperature difference between both areas on the sensor tip, low-temperature co-fired ceramics (LTCC) may be used as a sensor substrate to enhance the sensitivity significantly, owing to their lower thermal conductivity (ca. 2.5 W/mK, instead of 9 to 10 W/mK for alumina for 400 to 600 °C [20]). Unfortunately, the glass ceramic-based LTCC materials offer lower mechanical strength, which makes handling them difficult. It turned out that a combination of alumina with laminated glass-ceramic layers on the front side as substrate are a good compromise [21]. We used this technology for the Gen_2 sensors, in contrast to “blank” alumina substrates for the Gen_1 sensors.

On the reverse side of the substrate, a heater structure is applied (Pt, LPA-88, Heraeus) to heat the sensor to its operation temperature. Since it was made using a four-wire technique (Figure 1), it allowed us to establish a constant temperature by control to its four-wire resistance, measured particularly at the sensor tip, representing the heating zone. In all our investigations, we worked with 600 °C as the sensor tip temperature. This ensured, not only the activation energy to convert the reducing analyte gas species using the catalyst, but also to prevent deposits of soot or other particles (dusts) accumulating on the sensor’s surface. The heater is protected by the glass ceramic cover as well.

The sensor response behaves linearly with the concentration of the individual analytes. Hereby, not only the caloric values of the analytes, but also the reactivity and diffusion properties, determine the sensitivity. We found linear characteristics, but with individual slopes for each kind of analyte. The slope depends on the sensor setup (with all the above-mentioned factors for the sensing element), and also, on the ambience of the sensor such as housing, gas access through a protection cap, mounting position, etc. However, all these influences can be physically or chemically explained, so that model-based improvements and adaptions for specific applications are possible [22].

### 2.2. Sensor Housing

**Figure 2:**
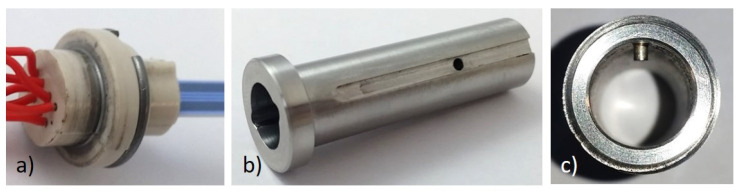
Developed sensor housing for defined horizontal sensor mounting as a modular setup: (**a**) two half shells (PEEK) clamp the ceramic sensor substrate together with electric contact wires; (**b**) a stainless steel adapter fits the flange (**c**), which is welded to the flue pipe of the chimney.

As mentioned before, the sensor periphery affects the access of the analytes to the functional layer. In our investigations, we worked with a special modular housing concept. Here, the ceramic sensing element is enclosed by two half-shells made of high-temperature, stable plastics (PEEK). Holes and notches are integrated in these half-shells to attach integrated hookup wires to the positions of the contact pads of the element. A clamping ring holds both half-shells together (Figure 2a). The sensor substrate has the only connection point with the housing at the location of the contact pads. An appropriate adapter (which might include a protective cap (Figure 2b)) is placed over the sensor element. The shape of the assembled half-shells precisely matches the adapter to hold the sensing element in a well-defined position relative to the cap. The cap comprises a nut, so that the installation position in the flange (Figure 2c) that is welded to the flue pipe in the chimney can be ensured with a corresponding pin. Such modular housing allows, amongst others, the rapid exchange of the sensing element and the use of different protective caps.

The development of such an improved housing concept was motivated by the need to thermally decouple the ceramic sensing element from the metal flanges and the environment to avoid heat transfer by conduction. In earlier investigations, we fixed the ceramic sensing element inside a stainless steel tube with a ceramic casting compound, which led to a weaker signal and less stability during the experiment. Furthermore, the new concept allowed us to ensure the defined horizontal mounting position of the sensing element, which is now arranged perpendicularly to the gas flow. The gas flow strongly affects the sensor signal since the cooling of the substrate is not equal over the entire sensor tip. This effect could be counteracted by positioning the sensing element in a laminar flow precisely perpendicularly, so that both areas are cooled simultaneously. Twisting it by a few degrees will also result in voltage changes [23].

Since the gas flow generally affects the temperature homogeneity of the sensor tip, the use of a protection cap around the sensing element is reasonable. Moreover, it will reduce the noise caused by the heater controller, and so, this enhances the signal stability. In all our experiments, we used a sintered metal filter element, which we applied to the correspondent adapter surrounding the sensor.

### 2.3. Positioning and Experimental Tests

**Figure 3:**
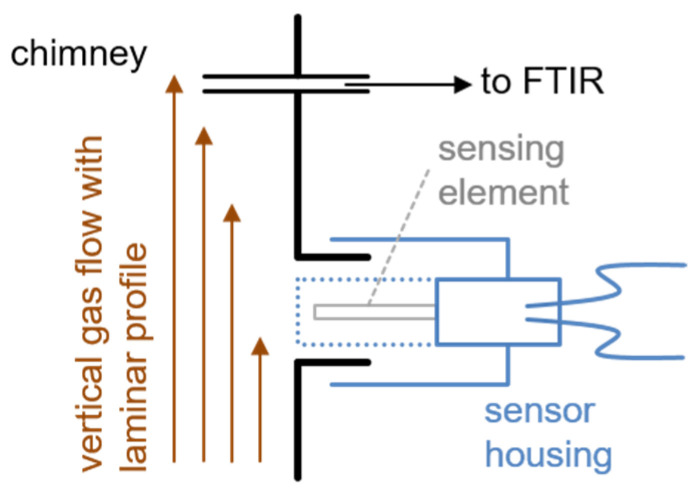
Scheme of the sensor mounting position in real exhaust measurements on the fringe of a vertical chimney. The tip of the sensing element is slightly recessed and not directly exposed to the gas flow.

Preliminary simulations of gas velocities in a suitable setup showed that a perpendicular mounting position on the exhaust pipe fringe of a vertical chimney could be advantageous. Despite there being a high exhaust mass flowing upwards inside the chimney, the gas velocity of its laminar flow in the boundary area, and so near the sensor tip, is minimal. Due to the mentioned protecting cap, the direct impact of a gas flow on the sensor tip is decreased significantly. Gas reaches the functional sensing layer by diffusion, which is sufficient concerning the response time in such applications. Figure 3 shows the setup with real, schematic exhaust measurements. Beside the sensor position, a gas probe is taken for continuous FTIR analysis (Gasmet, Ansyco GmbH, Karlsruhe).

For measurements in the Bayreuth lab, we chose a stainless steel chamber with a sensor mounting position that is similar to Figure 3 with a vertical gas flow mixed from cylinders. Real gas sensitivity tests performed with a simultaneous FTIR gas analysis were carried out in the wood firing technical center at ISIS in the flue gas of a fireplace (SF10SK, Brunner GmbH, Eggenfelden (Germany)).

## 3. Results

### 3.1. Proof of Concept (Gen_1)

For the preliminary test to check the sensor’s functionality in general and demonstrate its possible application in a flue gas analysis, we laid special focus on the high temperature stability and long-term stability of the sensor element. Therefore, the sensor setup as described above (denoted as Gen_1) was used, comprising an alumina substrate with Pt/PtRh thermopiles. The active layer was a 1 wt.-% Pt-loaded, porous alumina catalyst.

The sensor signal measured during a wood log charge combustion experiment is generated by a sum of reducing gas components in the flue gas, which oxidize when they reach the sensor tip. In addition to CO_2_ and water, the major pollutants produced during wood log combustion are CO and methane, but also, other unburnt hydrocarbons, hydrogen, and various other gas species (for example, HCHO) are typically emitted in lower concentrations. To evaluate whether the sensor reading images all the reducing components in the flue gas, the sensor signal is compared to gas concentration data from FTIR analytical flue gas analysis.

**Figure 4:**
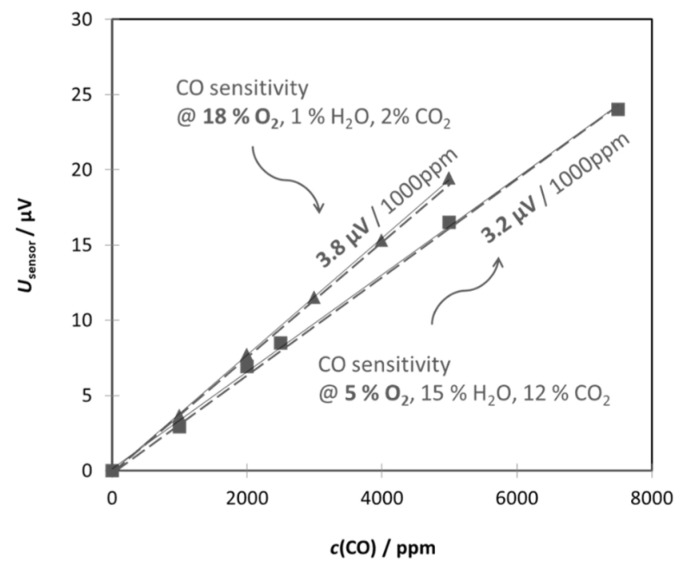
Typical characteristic dependency of the signal of a thermoelectric sensor; a linear relation between the sensor signal and the analyte is achieved. The characteristic is also affected by the base gas conditions such as oxygen concentration and humidity. The presented data of a Gen_1 sensor was derived in a lab measurement of synthetic gas without a protection cap.

As the sensor responds to different gas species with different sensitivities, we carried out lab tests with single test gases at first. Therefore, the sensors were installed in a measuring chamber in a similar position to the later chimney application. Synthetic exhaust gas flows were mixed from gas cylinders by mass flow controllers. Each test gas was admixed in increasing concentrations to a base gas that consisted of 10% O_2_ and humidified N_2_. The sensor characteristics are always linear (as visible in Figure 4 for Gen_1). For visualization and slope evaluation, the sensor’s offset voltage, which is measured without the test gas (0 ppm), was subtracted. The origin of such offset voltages might come from scattering effects during manufacturing or individuality in mounting. A detailed description of this theory and the facts derived from it are given in the discussion of the results.

During the transient combustion process of a wood log, huge fluctuations in the concentration of oxygen (ROC), CO_2_, or humidity in the flue gas are expected [7,8,9,10]. These “base gas” conditions could also affect the sensor performance. We investigated the CO sensitivity under typical “extreme” conditions (high O_2_/low H_2_O and low O_2_/high H_2_O). The difference in sensitivity changed by only about 10% between both “extreme” conditions. The reasons could be that less oxygen in the test atmosphere hinders the CO oxidation kinetics (Figure 4). The other measurements showed that even rough changes in the exhaust gas moisture can lead to changes in the sensor signal without reducing the test gas. However, this relationship has not yet been sufficiently clarified. As the concentrations of the three components, O_2_, CO_2_ and H_2_O, correlate with each other, one might correct the sensor signal “on-line”, e.g., by regarding the simultaneously collected secondary signals. Below, we introduce a weight factor on the basis of the monitoring of ROC by a lambda probe.

Different sensitivities toward different gas species are reflected in different slopes of such lab measurements. They depend not only on the caloric values of the gas molecules, which are catalytically converted at the sensors functional layer, but also, diffusivity and reactivity play a role in the transport limited process of the target gas reaction with oxygen, as already discussed for a caloric sensor chip [16], which is based on similar gas reaction processes. For instance, unsaturated hydrocarbons (such as, e.g., propene, C_3_H_6_) lead to a much larger response than its corresponding saturated species (propane, C_3_H_8_) does. For propane, the slope is just a little more than two times steeper than the slope of CO, and propene causes an effect that is around six times larger than the CO response is. However, in case of stable methane (CH_4_) exposure, the sensitivity is only half that of CO. More details on that are given in [22,24].

Basically, all the reducing gas components contribute additives to the signal with their individual caloric value and reaction rate on the functional layer. To correlate the sensor data with the FTIR analytic data during a combustion cycle of a wood log, we analyzed the lab findings concerning the sensor characteristics. From the evaluated slopes for different gas species, we derived weighting factors normalized to the CO response to form a sum of weighted reducing gas components referenced by the FTIR data as a CO-equivalent value to be represented by the overall sensor response during a charge combustion. This procedure is explained in the following text in more detail.

FTIR-measured concentration values for multiple gas species (*c*_i_) were summed up to obtain a collective sum concentration, whereby this summation considers the sensor-specific weighting factors (*z*_i_), which are derived from the slopes of the characteristic curves of individual gas species from the lab measurements (Equation (1)).

(1) $$c\left({\mathrm{CO}}_{e}\right)=\sum_{i}\mathrm{wt}\left(O_{2}\right)*z_{i}*c_{i}=\mathrm{SUM}\left({\mathrm{CO}}_{e}\right).$$

Since CO is the major component in the flue gas, the data are normalized to CO (i.e., weighting factor of CO is *z*_CO_ = 1), and the total sum of all the concentrations of the components contributing to the signal is called the “CO equivalent”, SUM(CO_e_). Unsaturated or long-chained hydrocarbons contribute more to the sensor signal, and therefore, are assessed with a higher factor. As already discussed above, the weighting factors depend not only on the combustion enthalpy of the individual component, but also on gas-specific parameters such as diffusivity and reactivity. The latter two parameters are also influenced by the sensor setup, mounting position or the use of special protection caps. In case of CH_4_, reactivity is low for Pt-loaded catalysts at 600 °C [24], so that its concentration is considered only with a factor of 0.5, for instance.

As mentioned above, also the ROC plays a role in the sensor response (see Figure 4). Therefore, we introduced a weighting factor wt(O_2_) in the range between 0 and 1, linearly depending on the ROC. For highly “lean” combustion, when a huge amount of oxygen is present in the flue gas (21% is the maximum value), the weighting factor is near the value of 1, so that all gas components are fully considered for *c*(CO_e_). At a lower ROC, wt(O_2_) decreases, respectively, so that the effect shown in Figure 4 is considered properly.

**Figure 5:**
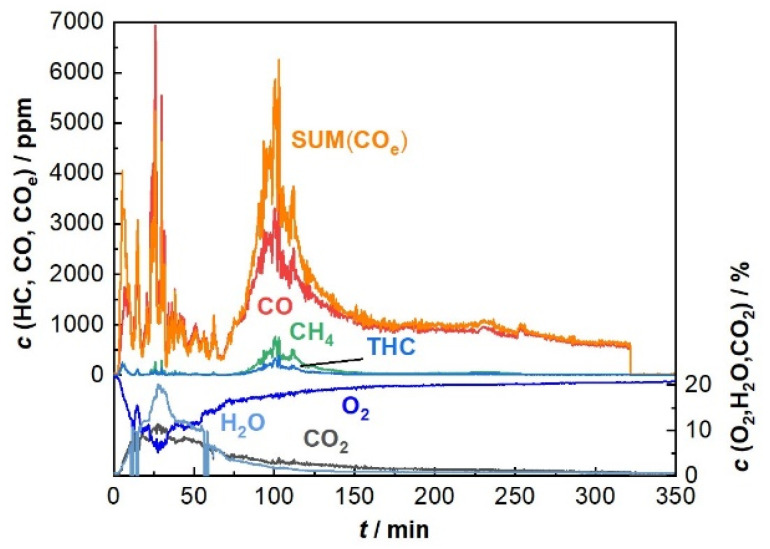
Typical emissions during charge combustion of a wood-log-fueled fireplace (HKD7, Brunner) measured by FTIR (only the main components such as CO, C_2_H_4_, CH_4_ and other hydrocarbons; THC plus CO_2_ and H_2_O are displayed). The ROC is logged using a commercial lambda probe (LSU 4.9, Bosch) and SUM(CO_e_) was calculated according to Equation (1).

Figure 5 shows an exemplary sum signal during charge combustion in a single room fireplace.

Here, one can see the difference between the course of *c*(CO) and that of SUM(CO_e_), which occurs if one considers the other main components, CH_4_ (*z*_CH4_ = 0.5), THC and C_2_H_4_ (*z*_C2H4_ = 13), whereby these fractions contribute by their special weighting factors (Equation (1)). Generally, CO has the main influence on the sensor signal. THC represents the “total hydrocarbons” as a typical FTIR measured value. For the sum evaluation, we chose to consider a mean value as weighting factor (*z*_THC_ = 5), which was determined using the values from the lab measurements. The SUM(CO_e_)-value was corrected by the secondary signal from the lambda sensor as well with its corresponding weighting factor wt(O_2_) over time. Many other components were determined by FTIR, but they occurred only in low concentrations (<100 ppm), and therefore, were not considered in the calculation of the sum signal.

**Figure 6:**
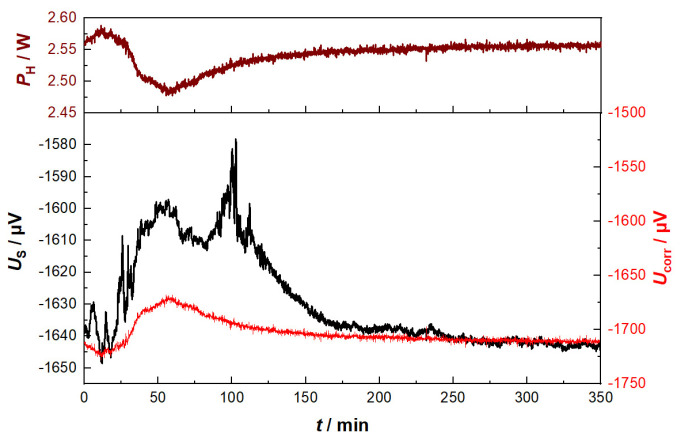
Sensor raw signal, *U*_s_, and heating power, *P*_H_, as calculated from the sensor heater data during the charge combustion cycle (corresponding flue gas analysis data are shown in Figure 5). From the heater power, an offset voltage was derived (*U*_corr_) to correct the raw data.

Beside these analytical data, the corresponding result (raw signal) of a Gen_1 sensor is given in Figure 6. At a first glance, the sensor signal shows a similar shape compared to that of the analytical data, but in the first 60 min, the sensor’s response is much larger.

A look at the heater power *P*_H_ might provide an explanation for this behavior. *P*_H_ decreases strongly during this time span and recovers until the end of the experiment. These findings coincide with the temperature changes in the exhaust and estimated temperature changes in the exhaust tube and sensor housing. Moreover, we see a correlation between the heater power and sensor signal. It is assumed that not only the catalytically caused temperature difference is affected here by secondary effects, but the sensors offset voltage changes too. Therefore, in the following considerations, the influence of these secondary effects induced by ambient temperature changes in the sensor’s offset voltage is discussed.

Scattering during manufacturing may cause an individual temperature distribution in the sensor tip area. This might affect the accuracy, as well as the positioning of the functional layers or the heater structure, i.e., when the layers are not aligned precisely to each other or in the middle of the substrate, especially regarding the heater structure. Edge effects (typical when one is using screen printing thin feed lines) will cause an individual distribution of the current densities in the Pt feeds of the meandered heater structure, and so, will cause temperature inhomogeneity in the heating zone. Thus, each sensor will have an individual offset voltage that remains constant at its operating temperature even without exothermic heat being generated at the catalyzed functional layer.

This offset voltage varies with the sensor operation temperature. Even changes in the heater power during control to achieve a constant operation temperature will affect this offset. Thermal loss by heat conduction, and also by convection, in a gas flow is compensated when one is controlling the heater to a constant four-wire resistance. So, changes in the local current density occur due to the above-mentioned scattering and have an influence on the temperature homogeneity. This might especially be affected by the sensor housing. Good thermal coupling between the sensing element and housing will result in increased heat transfer between them both. Therefore, the heater power (to keep the absolute temperature constant) is affected by the temperature changes in the housing. As the housing is directly connected to the exhaust tube by a flange, “cold start” conditions will cause huge changes in the heater power if the surrounding heats up. Hence, the surrounding influences will cause offset voltages that are no longer constant, but are affected by such secondary effects.

In a first step of data processing, the heater power (which is available as internal secondary data from the heater controller) was taken to calculate a corrected offset value of the sensor voltage as follows: Three points were identified, where SUM(CO_e_) is mostly zero, but *P*_H_ varies. These *P*_H_ data were linearly correlated with the measured sensor signal at these points. As SUM(CO_e_) should be zero here, the resulting function allowed us to derive a heater power-dependent offset curve that was valid for the entire experiment. The resulting offset curve (*U*_corr_) was subtracted from the sensor raw signal *U*_s_ to obtain a corrected sensor signal *U*_S,corrected_, which should show the response to reducing gases exclusively.

**Figure 7:**
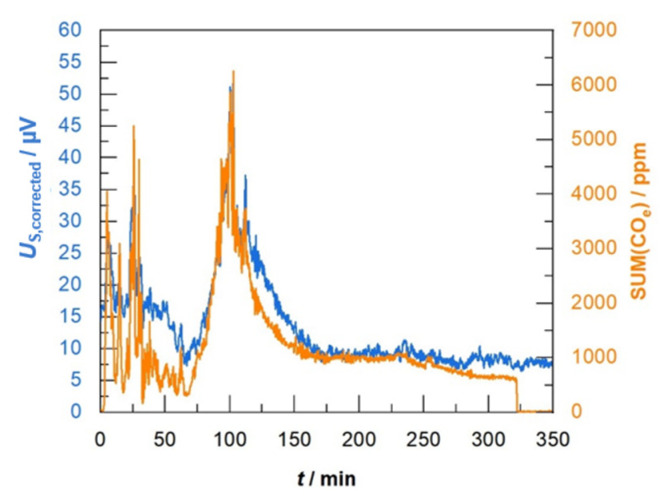
Corrected sensor signal (sensor type Gen_1) in comparison to analytic data (sum of CO_e_ measured by FTIR and weighted as described in the text).

The course of both data, the sensor signal *U*_S,corrected_ and SUM(CO_e_), are illustrated in one plot (Figure 7). There is an impressive correlation that can be observed. The response time of Gen_1 seems to be fully sufficient to indicate all the concentration peaks that occur during the different phases of the charge combustion process. Absolute values correlate also over the whole burning cycle. However, in the beginning of the experiment, the sensor data overestimated the CO_e_ concentration.

**Figure 8:**
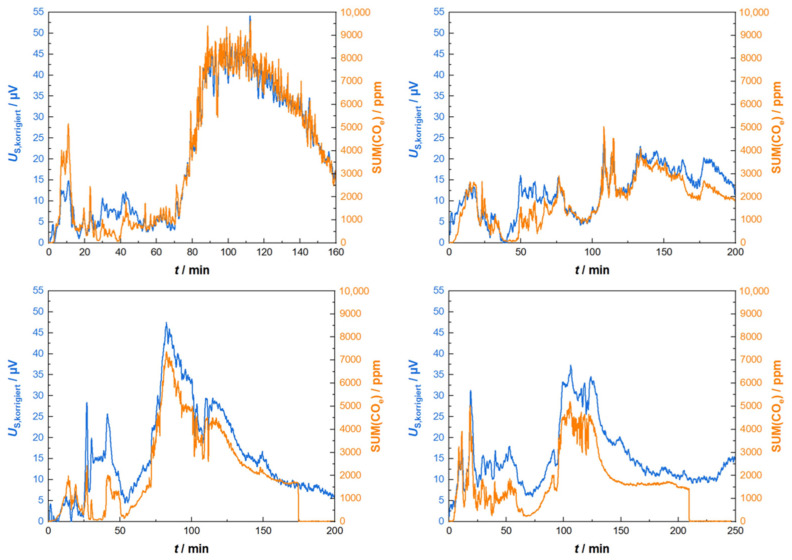
Corrected sensor signal (sensor type Gen_1) in comparison to analytic data for four different firing experiments. All raw signals were corrected with the same set of parameters as those described in the text and applied in the data in Figure 7.

Several other experiments (each starting with a single charge combustion cycle such as that described above) with Gen_1-sensors led to us obtaining the displayed curves in Figure 8. Hereby, the evaluations of SUM(CO_e_) were equal for all the experiments using the above given weighting factors for THC, CH_4_ and C_2_H_4_. Even more, the correcting function concerning the offset and heater power from the first experiment (Figure 7) was applied to all the other experiments. Discrepancies might occur due to the individual positioning of the sensor element, i.e., slightly twisted angles when remounting the sensor into the exhaust pipe of the exhaust tube.

Despite these inaccuracies between several real exhaust measurements, the sensor performance is good concerning the stability of its characteristic curve. It was tested in a lab atmosphere after each firing experiment to re-evaluate its response toward certain test gases (CO and H_2_). The sensor performance was found to remain stable after at least seven combustion cycles. These encouraging results justify further development efforts.

### 3.2. Next Generation Sensors: Sensitivity Enhancement (Gen_2)

Despite the first experiments with Gen_1 sensors being promising, several issues need to be development to enhance the sensor’s performance. Overall, the sensitivity should be further improved.

**Figure 9:**
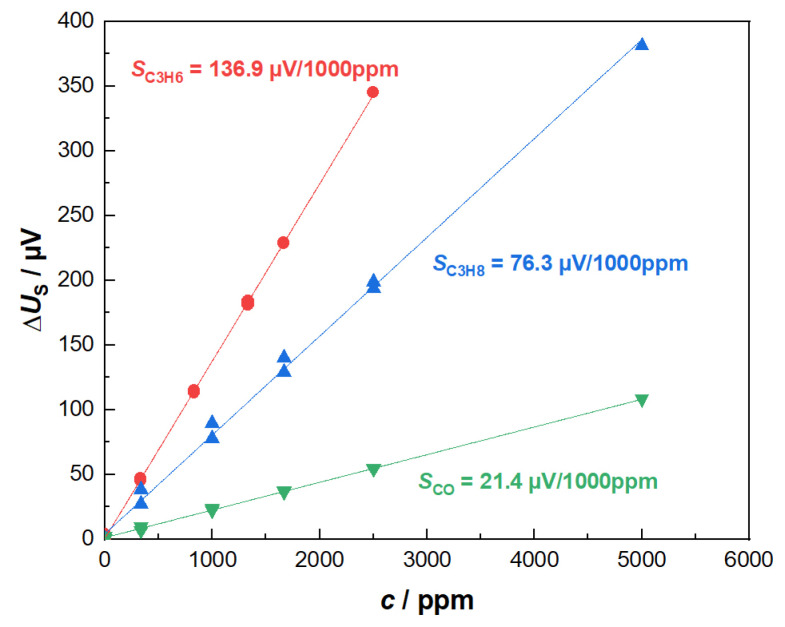
Characteristic sensitivity curves for Gen_2 sensors when they are exposed to model gases (base gas atmosphere: 10% O_2_ and 2% H_2_O).

As the process conditions (mainly the temperature of the exhaust tube and exhaust itself) at the location of the sensor remain always below 500 °C, and may therefore be regarded as moderate for the applied sensor materials, it is assumed that the sensor’s long-term stability will not be affected when other materials—such as Au as thermopile metal or feed line material—are used. Furthermore, an intermediate layer with a lower thermal conductivity was integrated, as already introduced in Section 2. Such a type of sensor (Gen_2) was installed as described above for several experiments in the lab and in a real exhaust as well. Again, for these experiments, a protection cap made of porous sintered metal was used. Lab data are displayed in Figure 9. The achieved characteristic curves show increased sensitivity (e.g., 5.6-fold increase for CO) in contrast to that of the Gen_1 sensor (see Figure 4). It should be noted here that these findings are transferable to other sensors with a similar setup.

The sensitivity factors derived from these measurements, together with the estimated values for calculating SUM(CO_e_), were very similar as those obtained before: *z*_CH4_ = 0.5, *z*_C2H4_ = 15 and *z*_THC_ = 5. The reproducibility concerning the manufacturing process of the sensing elements is high.

In the real exhaust measurements of wood log fueled firing experiments, expectably, the sensor signal must be corrected here also because there are still changes in the atmosphere with regard to the temperature of the exhaust tube and housing during the start, which could have affected the sensor signal.

**Figure 10:**
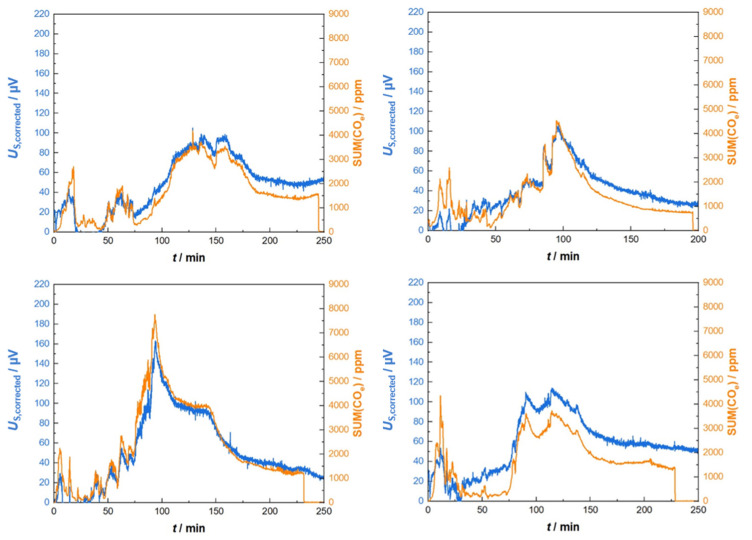
Sensor performance (Gen_2) during four different combustion cycles. Heating power correction and sum calculation was achieved with one parameter set such as that described in the text.

Therefore, again, we identified three points in time with similar gas conditions (vanishing amounts of reducing gases measured by FTIR), but at different heater power values. A linear relationship was derived again. This correction was then applied to all other measurements from Gen_2 of subsequent combustion cycles. Exemplarily, Figure 10 shows four different sets of sensor signals and FTIR data in direct comparison. The course of the corrected sensor signals and the corresponding CO_e_ data agree quite well, and the sensor’s responses reflected in the concentration peaks correspond nicely. Mostly, discrepancies occur in the beginning, where large variations in the flue gas flow, humidity changes and stronger variations in the heater power are unavoidable.

**Figure 11:**
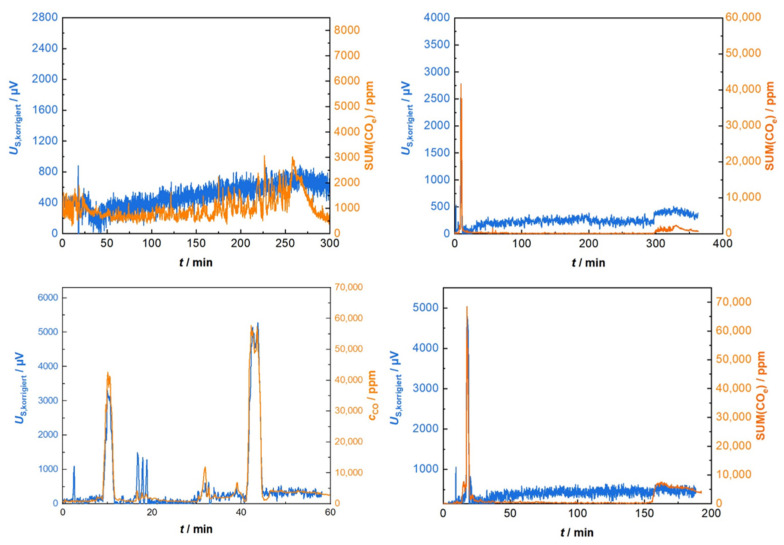
Sensor performance (Gen_2) when it was applied to a continuously operated biomass boiler (DBFZ, Leipzig). Heating power was corrected once before for all measurements. The sensor measures single peaks of emissions precisely up to 70,000 ppm.

These sensors were not only tested in the flue gas of single room furnaces, but also, in a continuously fueled biomass boiler at DBFZ (ÖKOTHERM^®^, Compact Biomass-Heating Systems Compact C0 with 49 kW, wood chips as fuel). Gas analysis data were collected simultaneously with the sensor data during firing as well. This allowed us to perform the calculation of the SUM(CO_e_) signal as described before (Equation (1)) to compare these data to the sensor response. Again, the heating power correction was determined only once. Figure 11 shows four experimental results. In contrast to former experiments in wood log fueled fireplaces with highly fluctuating emissions, the situation here is more stable, not only with regard to the emissions, but also with respect to the ambient conditions. Expectably, the sensor data agree well with the emissions values.

It is highly impressive that single peaks of emissions, which occur in the range up to 70,000 ppm CO during the cold start, malfunction and during the shutdown processes, are precisely measured by the sensor. Its linear characteristic curve seems to fit even with extreme conditions in such applications.

## 4. Conclusions and Outlook

Sensors for flue gas analyses will help to reduce the emissions released by wood log fueled fireplaces considerably by advanced automated combustion air stream control. In the last two decades, in several studies it has been discovered that besides the combustion temperature and residual oxygen concentration, also, the registration of the content of un- or partly combusted exhaust components by a high-temperature gas sensor plays a key role in obtaining improved descriptions of the combustion situation. However, such sensors must be able to withstand the harsh environment of hot emissions, which includes partially corrosive gas components, soot and particulate matter. Preliminary tests with thermoelectric gas sensors showed very promising results for measuring the CO equivalent of reducing gases in such exhausts. Its simple setup enables us to obtain a deep understanding of the sensing mechanism, and therefore, foster the future development of a cost-effective solution. As a matter of principle, the ambience conditions affect the sensor signal due to temperature inhomogeneities. Appropriate housing for the sensing element ensures defined and reproducible mounting and reduces the thermal coupling between the ceramic sensor tip and the ambient steel parts. Changes in temperature during the heating up of the exhaust tube and the sensor housing influence the required heater power of the sensor, and thus, affect the temperature distribution on the sensor tip and the sensor offset signal. A correlation between sensor offset and the heater power was found, and the offset voltages were corrected. The sensing element itself was significantly improved to enhance the sensitivity. Finally, an impressive correlation between the real gas analysis data and the sensor signals were found. It is expected that with this type of sensor, more exact control of the firing process will be achieved, which will lead to further emission reductions during biomass combustion processes.

Future work will focus on knowledge-based developments to reduce the cross-sensitivities. Simulation should include all the thermal processes that correlate with the changes in temperature homogeneity and help to refine the heater power with signal interaction. In conjunction with the development of a digital sensor electronic, these findings should lead to a real-time signal correction, preferably under consideration of more secondary data, such as ROC, or data from additional thermocouples regarding the exhaust or housing temperature.

Further developments regarding the sensing element might also address the integration of an oxygen sensor that is similar to the one as described in [25].

Experiments will focus on the sensors long-term stability. Furthermore, it should be investigated whether such types of sensors are applicable to other biomass use concepts as well, such as biomass waste combustion. Here, highly variable biomass fuel characteristics might cause very special needs for sensor stability. Therefore, tests on the impact of various special (corrosive) gas species arising in those exhausts, such as formaldehyde, or acid components, such as sulfur dioxide or hydrochloride, will be necessary.

Overall, a prospective sensor candidate was found that could help in reducing the emissions released by biomass combustion systems in the future.

## Data Availability

All relevant data presented in the article are stored according to institutional requirements and as such are not available online. However, all data used in this paper can be made available upon request to the authors.
